# A 3D architecture composite of porous vanadium nitride nanoribbons and reduced graphene oxide as a high-efficiency counter electrode for dye-sensitized solar cells†

**DOI:** 10.1039/c7ra11279c

**Published:** 2018-01-03

**Authors:** Guiqiang Wang, Shuo Hou, Chao Yan, Wei Zhang

**Affiliations:** School of New Energy, Bohai University Jinzhou 121013 China wgqiang@bhu.edu.cn

## Abstract

A three-dimensional (3D) porous architecture combining porous vanadium nitride nanoribbons with reduced graphene oxide was prepared through a hydrothermal process and subsequent thermal annealing in an ammonia/argon mixed atmosphere. Then, the obtained 3D porous vanadium nitride nanoribbon/reduced graphene oxide (PVNN/RGO) composite was explored as the counter electrode of dye-sensitized solar cells (DSCs). As evidenced by the electrochemical measurements, the 3D PVNN/RGO composite demonstrates excellent electrocatalytic performance, which is comparable to that of Pt. This can be attributed to the fact that the 3D architecture composite of porous vanadium nitride and reduced graphene oxide can simultaneously provide a favorable electrolyte diffusion channel, a fast electron-transport network, and an abundance of efficient electrocatalytic active sites. By employing such PVNN/RGO composite as the counter electrode, the fabricated DSC can achieve a conversion efficiency of 7.43%, which is comparable to that of the conventional Pt counter electrode (7.74%). Therefore, the 3D PVNN/RGO composite is a promising low-cost alternative to the expensive Pt as a counter electrode in DSCs.

## Abstract 2

3D PVNN/RGO composite electrode with hierarchical porosity exhibits superior photovoltaic performance competing with that of the conventional Pt electrode.

## Graphical abstract

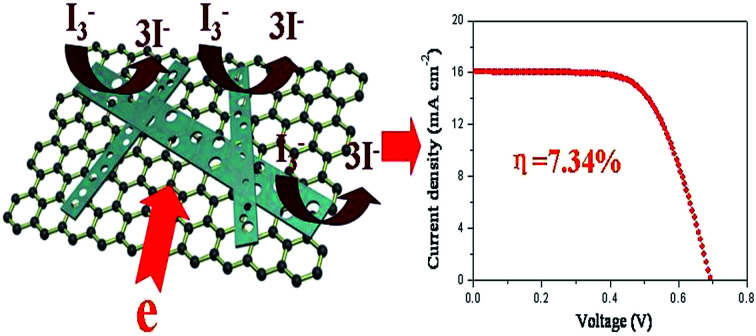

## 1 Introduction

Dye-sensitized solar cells (DSCs) have been extensively studied as one of the most promising alternatives to conventional Si solar cells due to their low cost, simple fabrication process, and high conversion efficiency.^1–4^ As one of the indispensable components in DSCs, the counter electrode plays two key roles: collecting electrons and reducing I_3_^−^ to I^−^ at the interface of the counter electrode and the electrolyte.^5^ Therefore, an ideal counter electrode material should exhibit superior electrocatalytic activity, high electrical conductivity, and good chemical stability. Thus far, Pt is the most widely used counter electrode material owing to its excellent electrical conductivity and superior electrocatalytic activity.^6–8^ However, the limited reserve and the high price are major hindrances for the large-scale application of Pt in DSCs. Consequently, several types of low-cost materials such as carbon materials,^9–12^ conducting polymers,^13–15^ transition metal compounds,^16–24^ and alloys^25,26^ have been developed to prepare Pt-free counter electrodes for DSCs. Some of them even exhibited superior photovoltaic performance to the conventional Pt counter electrode (see Table S1†).

Recently, transition metal compounds, in particular, have gained attraction as counter electrode materials because of their low cost, ease of modification, and simple fabrication.^16,27^ Of the transition metal compounds investigated, transition metal nitrides display excellent electrocatalytic activity for reducing I_3_^−^ to I^−^ due to their noble metal-like surface electronic structure and the energy level.^28–30^ However, it is not easy for transition metal compounds to simultaneously display good electrical conductivity and superior electrocatalytic activity, even though it is an essential requirement for high-performance counter electrodes in DSCs. In general, nanoparticles can provide good electrocatalytic activity, but they increase the electron transport barrier owing to the abundant particle boundaries.

Constructing an efficient electron transport network and developing electrocatalytic active sites on the electron transport pathway is a promising strategy to synergistically combine electrical conductivity and electrocatalytic activity in a single material.^31^ Graphene and carbon nanotubes (CNT) can be considered as appropriate materials for creating a fast electron transport network. Cui *et al.* prepared transition metal nitrides/N-doped graphene hybrids, in which MoN, TiN, and VN nanoparticles were dispersed on the surface of N-doped graphene.^32^ These hybrid materials displayed higher photovoltaic performance than Pt. This improved performance is attributed to the combination of high electrical conductivity of the carbon electron-transport network and excellent electrocatalytic activity of transition metal nitride nanoparticles. Gao *et al.* prepared TiN–CNT composite by anchoring TiN nanoparticles on the CNT network,^33^ which showed a comparable photovoltaic performance with Pt.

Herein, a 3D architecture that combines porous vanadium nitride (VN) nanoribbons with reduced graphene oxide (RGO) was prepared and investigated as a counter electrode for dye-sensitized solar cells. The 3D architecture results in the formation of the interconnected hierarchical pore structure, which provides a favorable channel for electrolyte diffusion and then enhances the accessibility of electrode material. The highly conductive RGO network improves the electrical conductivity, and the porous nanoribbon structure of VN increases the electrochemically active surface area. As a result, the porous VN nanoribbon/reduced graphene oxide (PVNN/RGO) composite electrode displayed an excellent electrocatalytic activity for the reduction reaction of I_3_^−^. The DSC based on the PVNN/RGO counter electrode achieved a conversion efficiency of 7.43%, which is comparable to that of the cell based on a Pt counter electrode.

## 2 Experimental

### 2.1 Preparation of 3D PVNN/RGO composite

NH_4_VO_3_ (0.4 g) was dissolved in a 100 mL mixture of water and ethanol with a volume ratio of 9 : 1. Then, the pH value of this solution was adjusted to 3 by adding HCl. Graphene oxide (GO) (0.2 g) was dispersed in 100 mL of deionized water through sonication. Following this, the NH_4_VO_3_ solution was mixed with the GO dispersion by stirring at room temperature. The volume ratio of the NH_4_VO_3_ solution to the GO dispersion was 1 : 1. The above mixture was then transferred to a Teflon-lined autoclave and heated at 180 °C for 24 h. The as-prepared samples were rinsed with deionized water for several times and freeze-dried for 48 h. Then, the freeze-dried samples were annealed at 600 °C for 3 h in NH_3_/Ar (40 sccm:40 sccm) mixed gas. For comparison, pure VN was prepared *via* the same procedure, but without GO. As shown in Fig. S1,† the as-prepared pure VN displays the form of porous nanoribbons.

### 2.2 Preparation of PVNN/RGO electrodes and fabrication of DSCs

PVNN/RGO electrodes were prepared by spraying the PVNN/RGO dispersion on pre-cleaned F-doped tin oxide (FTO) glass and subsequent treatment at 350 °C for 20 min under nitrogen atmosphere. The PVNN/RGO dispersion was prepared by grinding 200 mg of PVNN/RGO with 15 mL of isopropanol, 0.1 mL of 20 wt% Triton X-100 aqueous solution, and 20 μL of tetrabutyl titanate in a mortar, followed by ultrasonication for 30 min.

For the fabrication of TiO_2_ photoanodes, a TiO_2_ colloidal paste was prepared by mixing TiO_2_ (P25), Triton X-100, and hydroxypropyl cellulose in a mixed solution of deionized water and isopropanol with a volume ratio of 3 : 7. Then, the TiO_2_ electrodes were prepared by coating the TiO_2_ colloidal paste on FTO glass using the doctor-blade method and subsequent sintering at 450 °C for 30 min. The thickness of the TiO_2_ film was about 9 μm. Then, the as-prepared TiO_2_ electrodes were immersed in a 0.5 mM ethanol solution of N719 dye at room temperature for 16 h. Finally, the dye-sensitized TiO_2_ electrodes were rinsed with absolute ethanol. A DSC was fabricated by assembling a dye-sensitized TiO_2_ electrode, the electrolyte, and a counter electrode in a sandwich structure. The electrolyte was composed of 0.3 M LiI, 0.5 M 1-hexyl-3-methylimidazolium iodide, 0.05 M I_2_, and 0.4 M 4-*tert*-butylpyridine with 3-methoxyproponitrile as the solvent.

### 2.3 Characterization and measurements

The as-synthesized materials were characterized using scanning electron microscopy (SEM, Hitachi S-4800), X-ray diffraction (XRD, Bruker D8 Advance Cu K_α_), and Raman spectroscopy (Renishaw Invia, the excitation wavelength of 532 nm). N_2_ sorption measurements were carried out at 77 K using a Micromeritics ASAP 2020 physisorption analyzer. Electrochemical impedance spectroscopy (EIS) measurements were carried out using a Solartron 1287/1255 electrochemical system with an amplitude of 10 mV in the frequency range from 10^5^ Hz to 0.1 Hz. Tafel polarization measurements were performed using dummy cells. The electrolyte used in dummy cells was as same as that used in DSCs. The photocurrent density–voltage characteristics of DSCs were tested using a Keithley 2400 source meter under AM 1.5 illumination at a light intensity of 100 mW cm^−2^.

## 3 Results and discussion

**Fig. 1:**
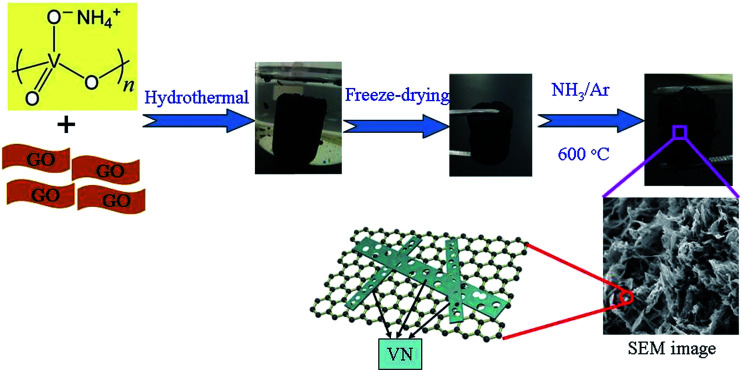
Schematic diagram for preparing the 3D PVNN/RGO composite.

**Fig. 2:**
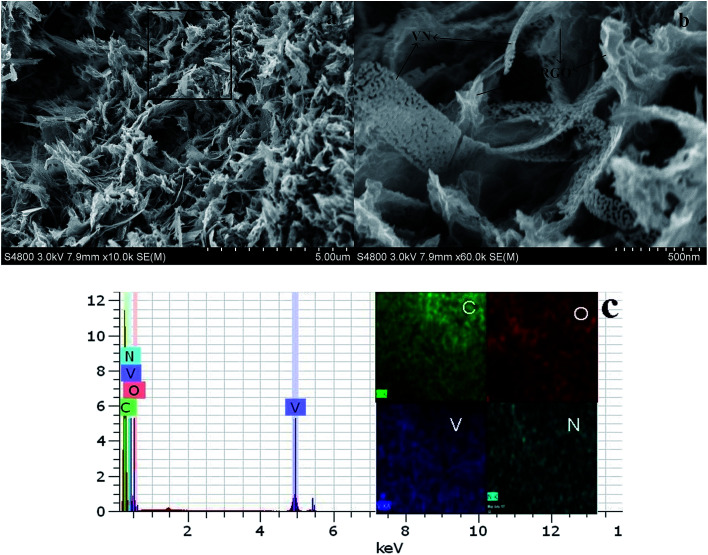
(a, b) SEM images of the as-prepared 3D PVNN/RGO composite, (c) energy dispersive X-ray spectrum and elemental mapping for a rectangular area indicated in (a).

The 3D PVNN/RGO composite was prepared by a simple hydrothermal process using GO and NH_4_VO_3_ as the precursors, followed by annealing in an NH_3_/Ar mixed atmosphere at 600 °C (as shown in Fig. 1). The perfect 3D porous architecture of PVNN/RGO composite is formed when the volume ratio of NH_4_VO_3_ solution and GO dispersion is 1 : 1. Therefore, this sample was selected and investigated below. During the hydrothermal process, GO was reduced^34^ and NH_4_VO_3_ was decomposed into vanadium oxide, which leads to the formation of a 3D vanadium oxide nanoribbons/reduced graphene oxide composite. As shown in Fig. S2,† vanadium oxide nanoribbons interpenetrate with reduced graphene oxide nanosheets to form a porous composite. Vanadium oxide nanoribbons transform into porous VN nanoribbons through annealing in the NH_3_/Ar atmosphere. Fig. 2a and b show the SEM images of the as-prepared PVNN/RGO sample. SEM images of the PVNN/RGO composite clearly indicate the uniform embedment of porous VN nanoribbons in the graphene layers to form the 3D interpenetrating architectures. The dimensions of the VN nanoribbons are in the range of 100–200 nm in width and several micrometers in length. As shown in Fig. 2c, the energy dispersive X-ray spectrum confirms the composition of the as-prepared composite that consists of V, N, C, and O. The elemental mapping on the area, indicated in Fig. 2a, demonstrates that VN is distributed homogeneously over the graphene layer.

**Fig. 3:**
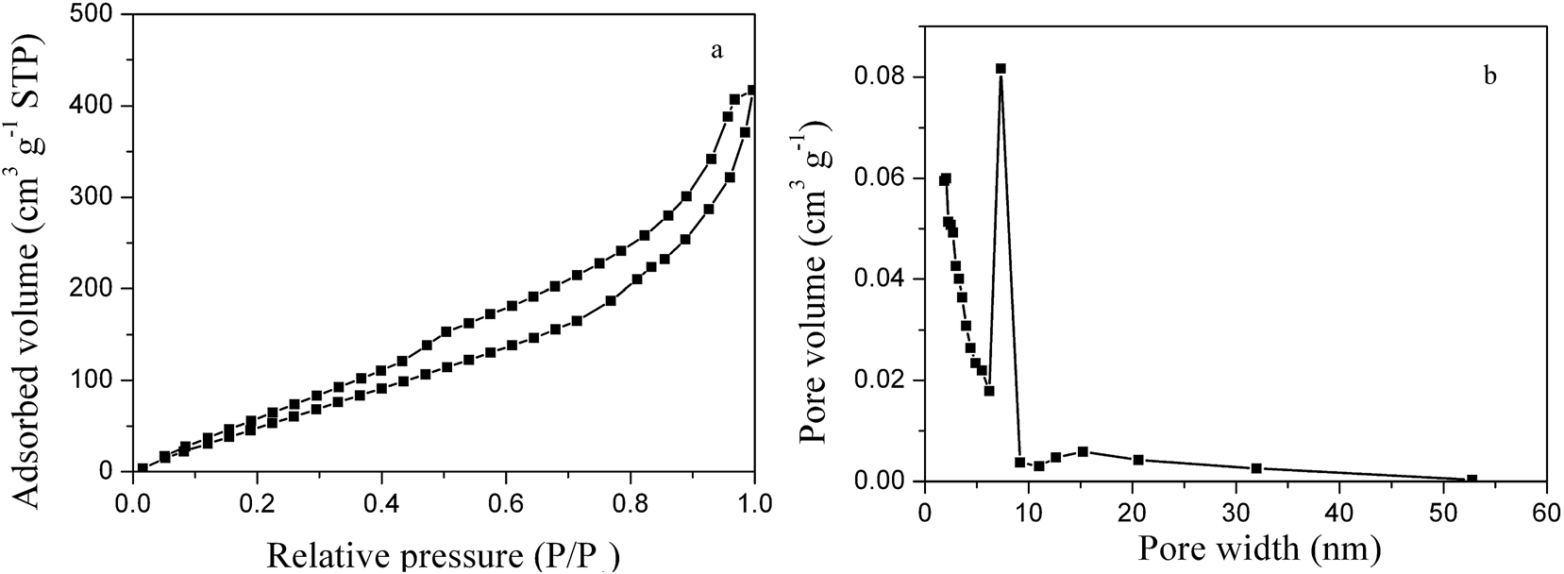
N_2_ adsorption–desorption isotherms (a) and pore-size distribution curve (b) of the 3D PVNN/RGO composite.

The Brunauer–Emmet–Teller (BET) surface area and the pore structure of the as-prepared 3D PVNN/RGO composite were determined by N_2_ adsorption measurement at 77 K. Fig. 3 displays the N_2_ adsorption–desorption isotherm and the pore-size distribution curve of the PVNN/RGO sample, which clearly suggest that the as-prepared PVNN/RGO composite possesses a hierarchical porous structure consisting of small mesopores (2–10 nm) and large mesoporous (10–50 nm). The BET surface area of the PVNN/RGO composite calculated from the desorption branch of the isotherms is 200.5 m^2^ g^−1^, which is higher than that of the pure VN sample (Fig. S3†). High surface area and the hierarchical porosity can increase the effective electrode/electrolyte interface and enhance the electrolyte diffusion within the electrode material, which are favorable characteristics for improving the electrochemical performance of the electrode material.

**Fig. 4:**
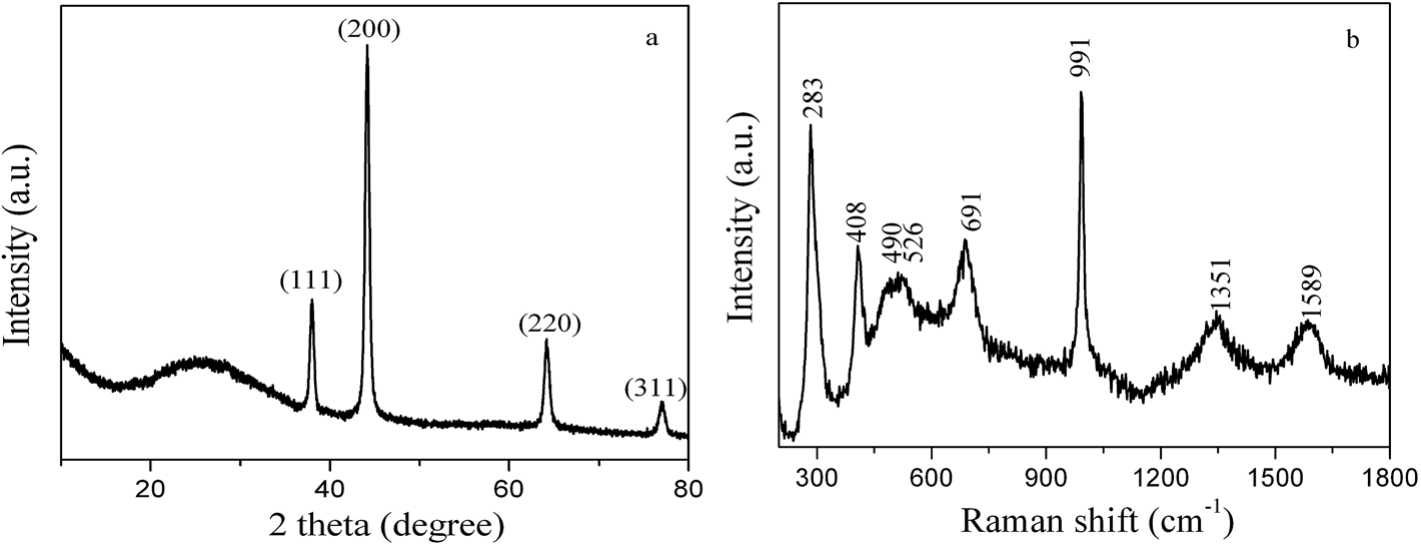
XRD pattern (a) and Raman spectrum (b) of the as-prepared 3D PVNN/RGO composite.

Fig. 4a shows the XRD curve of the as-prepared 3D PVNN/RGO composite. A broad peak at 2*θ* value of around 26° can be assigned to graphene stacking. Four strong diffraction peaks at 2*θ* values of 38.08°, 44.18°, 64.18°, and 77.06° are ascribed to the crystal planes of (111), (200), (220), and (311) of cubic VN (JCPDS card no. 73-0528), respectively. This indicates that vanadium oxide obtained by thermal decomposition of NH_4_VO_3_ transformed into VN. Raman spectroscopy was employed to further investigate the structural information of the 3D PVNN/RGO composite. Fig. 4b shows the Raman spectrum of the as-prepared 3D PVNN/RGO composite. The two peaks observed at around 1351 and 1589 cm^−1^ correspond to the characteristic D and G bands of graphene, respectively. The peaks at 283, 408, 490, 526, 691, and 991 cm^−1^ can be attributed to the typical modes of vanadium oxide, indicating that a tiny amount of vanadium oxide was formed on the surface of VN nanoribbons. Although the formed vanadium oxide layer was too small to be detected by XRD (see Fig. 4a), the vanadium oxide layer on VN surface exhibits a very well resolved Raman signature, which dominates the Raman spectrum due to the very low intensity of VN peaks.^35^

**Fig. 5:**
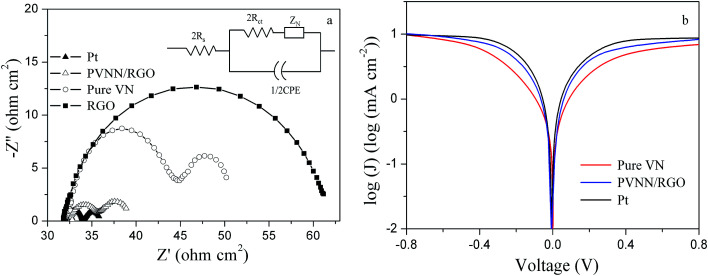
(a) Nyquist plots of the cells fabricated with pure VN, RGO, PVNN/RGO, and Pt electrodes, (b) Tafel curves of pure VN, PVNN/RGO, and Pt electrodes.

The PVNN/RGO composite was deposited on a pre-cleaned FTO glass substrate by a spraying method. Electrocatalytic activity of the PVNN/RGO electrode for the reduction of I_3_^−^ was investigated by EIS measurements using symmetric cells consisting of two identical electrodes. Fig. 5a shows the Nyquist plot of the PVNN/RGO electrode. For comparison, Nyquist plots of pure VN, RGO, and conventional Pt electrodes are also shown in Fig. 5a. As shown in Fig. 5a, Nyquist plots exhibit two semicircles, which are attributed to the Nernst diffusion impedance of the I^−^/I_3_^−^ redox couple within the electrolyte and the charge-transfer process at the electrode/electrolyte interface in the order of increasing frequency. The inset in Fig. 5a is the equivalent circuit for the impedance spectra, where *R*_s_ is the serial ohmic resistance, *R*_ct_ is the charge-transfer resistance, CPE is the constant phase element, and *Z*_N_ is the Nernst diffusion impedance. EIS parameters (listed in Table 1) were determined by fitting Nyquist plots with Zview software. The *R*_s_ values of all the electrodes are primarily dominated by the resistance of FTO substrate. Therefore, the *R*_s_ of pure VN, RGO, and PVNN/RGO electrodes are comparable to that of the Pt electrode. The CPE of the PVNN/RGO electrode is noticeably higher than that of the pure VN electrode, indicating a higher active surface area of PVNN/RGO compared to pure VN. The *R*_ct_ is a vital and widely used parameter to evaluate the electrocatalytic activity of the counter electrode for the reduction of I_3_^−^. As listed Table 1, the *R*_ct_ values of Pt, PVNN/RGO, pure VN, and RGO electrodes are 1.02, 1.62, 8.12, and 15.21 Ω cm^2^, respectively. Clearly, the electrocatalytic activity of the PVNN/RGO electrode is higher than that of pure VN and RGO electrodes in the I^−^/I_3_^−^ electrolyte. The enhanced electrocatalytic performance of the PVNN/RGO electrode can be attributed to its unique 3D porous architecture resulting from the combination of porous VN nanoribbons with RGO nanosheets. The 3D porous architecture can provide an interconnected channel for the fast diffusion of the electrolyte. Highly conductive RGO network is favorable for electron transport, and porous VN nanoribbons provide a higher effective surface area. The combination of these favorable features can greatly enhance the electrocatalytic performance of the PVNN/RGO electrode.

**Table 1:** Electrochemical parameters of Pt, pure VN, RGO, and PVNN/RGO electrodes and the photovoltaic parameters of DSCs with various counter electrodes

Electrode	*R*_s_ (Ω cm^2^)	*R*_ct_ (Ω cm^2^)	CPE (μF)	*V*_oc_ (V)	*J*_sc_ (mA cm^−2^)	FF	*η* (%)
Pt	15.95	1.02	7	0.71 ± 0.01	16.52 ± 0.22	0.66 ± 0.01	7.74 ± 0.30
Pure VN	16.17	8.12	65	0.69 ± 0.01	15.66 ± 0.21	0.58 ± 0.02	6.27 ± 0.39
PVNN/RGO	16.18	1.62	326	0.71 ± 0.01	16.09 ± 0.25	0.65 ± 0.01	7.43 ± 0.33
RGO	16.31	15.21	32	0.64 ± 0.02	13.59 ± 0.25	0.38 ± 0.02	3.32 ± 0.34

Tafel polarization measurements were conducted to further assess the electrocatalytic activity of the PVNN/RGO electrode. Fig. 5b displays the Tafel curves of dummy cells fabricated with pure VN, PVNN/RGO, and Pt electrodes. Tafel curve can be divided into three regions: the polarization zone, the Tafel zone, and the diffusion zone. In the Tafel zone, the exchange current density can be determined, which is directly related to the electrocatalytic activity of the electrode. Fig. 5b exhibits that the slope of the cathodic branch for the PVNN/RGO electrode is larger than that for pure VN electrode and comparable to that of the Pt electrode, indicating a higher exchange current density on the surface of the PVNN/RGO electrode. This result demonstrates that the PVNN/RGO electrode can catalyze the I_3_^−^ reduction as effectively as the Pt electrode, which is in good agreement with the EIS result.

**Fig. 6:**
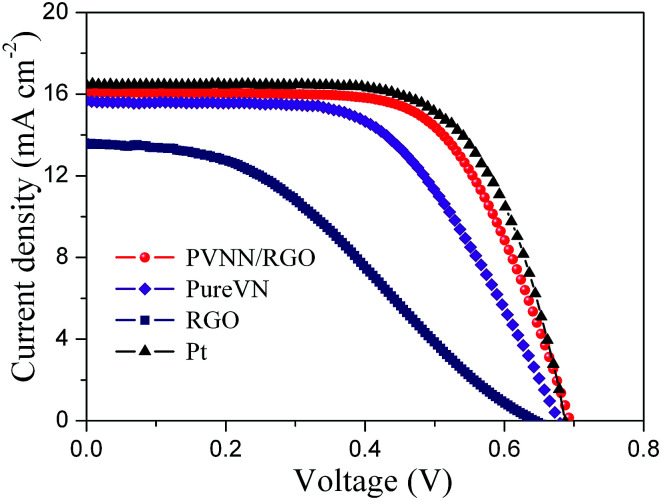
Photocurrent density–voltage curves of DSCs with different counter electrodes measured at AM 1.5G illumination (100 mW cm^−2^).

The photovoltaic performances of DSCs fabricated by PVNN/RGO, pure VN, RGO and Pt counter electrodes were obtained under an illumination of 100 mW cm^−2^. The current density–voltage curves are depicted in Fig. 6. The photovoltaic parameters of DSCs derived from Fig. 6 are summarized in Table 1. The RGO counter electrode exhibits a low conversion efficiency of 3.32% due to its poor electrocatalytic activity. The open-circuit voltage (*V*_oc_), the short-circuit current density (*J*_sc_), the fill factor (FF), and the conversion efficiency (*η*) of the DSC assembled with the PVNN/RGO counter electrode are 16.09 mA cm^−2^, 0.71 V, 0.65, and 7.43% respectively. When pure VN was used as the counter electrode, the fabricated DSC showed a lower *η* of 6.27%, primarily due to a lower FF of 0.58. As we know, the FF is strongly influenced by the internal resistance of the DSCs. For DSCs fabricated with different counter electrodes, the *R*_ct_ value of the counter electrode has a great effect on the FF of DSCs. As shown in Table 1, the *R*_ct_ value of the PVNN/RGO electrode is measured to be lower than that of the pure VN electrode. Therefore, the PVNN/RGO counter electrode-based DSC has a higher FF. The photovoltaic parameters of the DSC with the Pt counter electrode are *V*_oc_ = 0.71 V, *J*_sc_ = 16.52 mA cm^−2^, FF = 0.66, and *η* = 7.74%. Clearly, the photovoltaic performance of the DSC with the PVNN/RGO counter electrode is comparable to that of the DSC with the Pt counter electrode.

## 4 Conclusions

A 3D PVNN/RGO composite was prepared by the combination of a hydrothermal process and thermal annealing in NH_3_/Ar atmosphere, and it was investigated as a high-performance counter electrode material for DSCs. Due to the unique 3D porous architecture, the as-prepared PVNN/RGO composite displays superior electrocatalytic activity for the reduction of I_3_^−^. Under illumination of 100 mW cm^−2^, the DSC based on the PVNN/RGO counter electrode achieved a conversion efficiency of 7.43%, which is close to that of the cell based on the Pt counter electrode. This study presents a promising method to fabricate Pt-free materials for the high-performance counter electrode of DSCs.

## Conflicts of interest

There are no conflicts to declare.

## Supplementary Material

RA-008-C7RA11279C-s001
