# Supplementary material for: A 3D architecture composite of porous vanadium nitride nanoribbons and reduced graphene oxide as a high-efficiency counter electrode for dye-sensitized solar cells
Source: RSC Adv. 2018 Jan 3;8(2):1083–8. doi: 10.1039/c7ra11279c (PMC9077002; doi:10.1039/c7ra11279c)
Supplement: RA-008-C7RA11279C-s001 [file RA-008-C7RA11279C-s001.pdf]

Electronic Supplementary Material (ESI) for RSC Advances.
This journal is © The Royal Society of Chemistry 2017

Supplementary material

3D porous vanadium nitride nanoribbon/reduced oxide graphene composite as a high-efficiency counter electrode for dye-sensitized solar cells

Guiqiang Wang [a], Shuo Hou [a], Chao Yan, Wei Zhang [a]

[a] School of New Energy, Bohai University, Jinzhou 121013, China

Table S1 Photovoltaic parameters of DSCs with metal selenide counter electrodes

Counter electrode	V_{oc} (V)	J_{sc} (mA cm^{-2})	FF	η (%)	Ref.
$Co_{0.85}Se$	0.738	16.98	0.75	9.4	[22]
Pt	0.738	16.03	0.73	8.64	
$CoSe_2$	0.753	18.55	0.73	10.20	[S1]
Pt	0.724	15.89	0.71	8.17	
$NiSe_2$	0.734	15.94	0.74	8.69	[23]
	0.731	15.26	0.72	8.04	

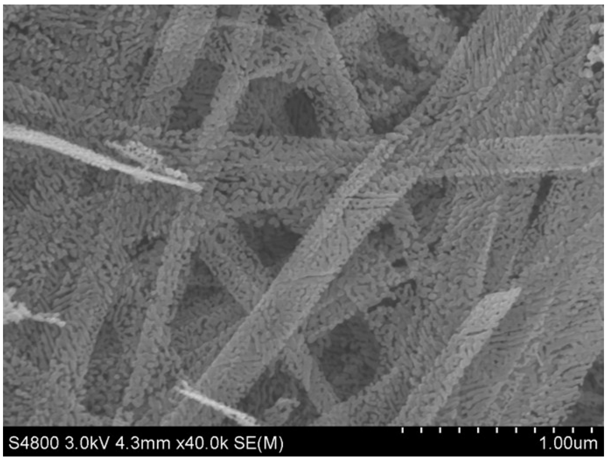

Fig. S1 SEM image of as-prepared pure VN

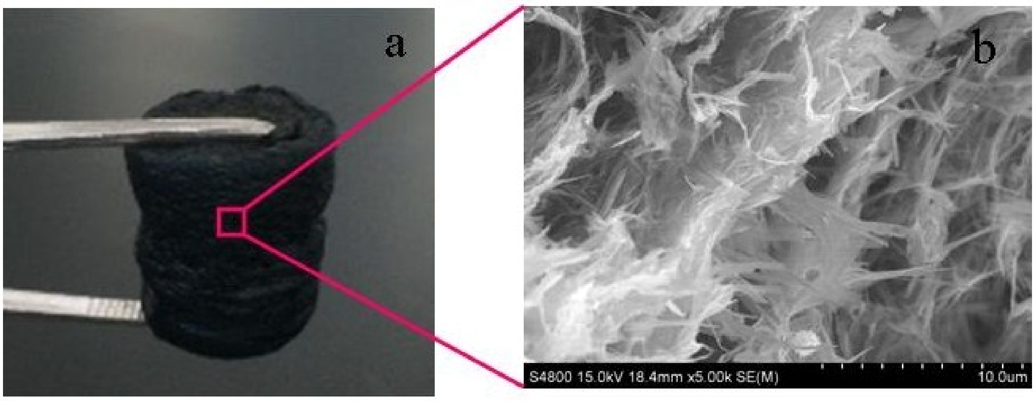

Fig. S2 Photograph (a) and SEM image (b) of as-prepared vanadium oxide nanoribbons/reduced graphene oxide composite.

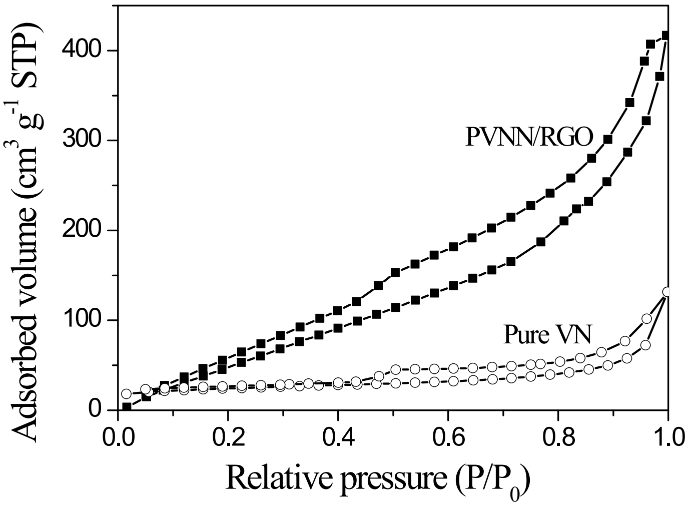

Fig. S3 Nitrogen Adsorption-desorption isotherms of PVVN/RGO and pure VN samples. The BET surface area of as-prepared pure VN determined from desorption branch is 80.7 m^2 g^{-1}.

References

[S1] H. Sun, L. Zhang, Z. Wang, J. Mater. Chem. A, 2014, 2, 16023.
